# New Appliances and Things Medical

**Published:** 1906-10-13

**Authors:** 


### NEW APPLIANCES AND THINGS MEDICAL.

[We shall be glad to receive at our Office, 28 & 29 Southampton Street, Strand, London, W.C., from the manufacturers, specimens of all new preparations and appliances which may be brought out from time to time.]

### CEREBOS SALT.

(Cerebos, Ltd., 3 Maiden Lane, London, E.C.)

This famous table salt has been submitted to us for examination, and, it is needless to say, maintains its qualities for digestibility and savour. Besides the chloride of sodium this preparation contains phosphates analagous to those present in the natural fluids of the body, as indispensable for the processes carried on in the blood and in the muscles of the body. The combination of the salts of sodium with phosphates renders Cerebos not only a flavouring ingredient, but a food in itself. This compound salt is particularly suitable for children. It is economical, and when used instead of common salt in bread-making, adds to the phosphatic value of the bread. Its preparation has been gradually within the last ten years brought to a very high state of perfection. It has an elegant appearance, white and granular, like sifted sugar, and is certainly one of the daintiest and most highly prized articles on the table. For travellers in warm climates it is most useful on account of its not readily clogging. It is protective also to the bony structures of the body, and prevents the decay of teeth. Further, it is well known that frequent pregnancy causes in many instances rapid decay of the teeth. This is due to a loss of phosphates and calcium salts arising from the drain in the system of these substances towards the formation of the bony structures in the offspring. Cerebos salt is an excellent means of making good this loss, and its use continuously in the food of pregnant women will tend to harden the enamel of the teeth and to prevent caries, and so may certainly prove of special value in suitable cases.

### THE "EUREKA" CREPE VELPEAU BANDAGE.

(Victoria House, Albion Place, Blackfriars Bridge, S.E.)

This bandage is designed to accomplish the purpose formerly served by the rubber elastic bandage, the disadvantage of which was due to the want of porosity and to the tendency to set up heat and inflammation at the point where support was required just at the time when coolness and soothing were needed. Many persons were prevented from using the rubber bandage on that account. This crêpe bandage stretches as readily as an elastic one, although no elastic enters into its construction. On testing its distensibility it is found to stretch more than twice its own measurement—the nine-foot bandage easily extending to six yards when applied to a limb. It is comfortable and light. It is easily cleansed, and can thus be used over and over again, and is not injured by washing. Nothing can be more comforting to an inflamed varicose limb than this bandage when carefully applied. For dressings for the scalp and face and those parts of the body which present difficulties these rubberless elastic bandages will prove a veritable blessing, and are almost indispensable in any considerable surgical practice. For those persons whose duties require them to be much on their legs, or whose feet have a tendency to swell, they can be applied underneath the stocking in the manner of putties, and will then afford an effective and comforting support.

